# A Critical Theoretical Approach to Cancer Disparities: Breast Cancer and the Social Determinants of Health

**DOI:** 10.3389/fpubh.2021.674736

**Published:** 2021-05-21

**Authors:** Sarah Gehlert, Darrell Hudson, Tina Sacks

**Affiliations:** ^1^Suzanne Dworak-Peck School, University of Southern California, Los Angeles, CA, United States; ^2^Brown School, Washington University in St. Louis, St. Louis, MO, United States; ^3^School of Social Welfare, University of California, Berkeley, Berkeley, CA, United States

**Keywords:** cancer, breast, social determinants, poverty, race, health disparities

## Abstract

Breast cancer is the most commonly experienced cancer among women. Its high rates of incidence and survival mean that a number of women will live it for periods of their lifetimes. Group differences in breast cancer incidence and mortality occur by race and ethnicity. For example, while white women are slightly more likely to be diagnosed with breast cancer, Black women are 40% more likely to die from the disease. In this article, rather than focusing the discussion on individual-level factors like health behaviors that have the potential to blame Black women and those living in poverty for their conditions, we view breast cancer disparities through the lens of Critical Race Theory, taking a historical perspective. This allows us to delve beyond individual risk factors to explore social determinants of breast cancer disparities at the population level, paying special attention to the myriad ways in which social factors, notably views of race and discriminatory public policies, over time have contributed to the disproportionate breast cancer mortality experienced by Black women. We suggest ways of addressing breast cancer disparities, including methods of training healthcare professionals and public policy directions, that include rather than marginalize Black and lower socioeconomic status women.

## Introduction

The term “cancer” refers to a group of diseases sharing significant characteristics such as the rapid proliferation of cells. Yet this group of diseases, referred to as cancer types, varies in a number of ways that affect their impact on individuals experiencing them and their social networks and communities. Because cancer types vary by site of origin (e.g., prostate, breast, colon, pancreas, and blood), require more or less demanding and costly treatment approaches, and have markedly different incidence, mortality, and survival rates, it is problematical to consider cancer as a single entity in determining how it affects, and is affected by, an individual's social circumstances. In the following manuscript, we focus on breast cancer, the most commonly experienced cancer among women worldwide. According to the National Cancer Institute, 279,100 women were diagnosed with breast cancer in the United States in 2020, making it an area worthy of attention (1).

Prevention and treatment of cancer depend on knowledge of its determinants and their interplay. Arguably, social determinants have received less attention than have genetics and individual health behaviors. After briefly outlining what is known about the social determinants of breast cancer disparities and drawing on salient theory, we provide our perspective on the social contributors to disparities. We suggest new directions for the training of healthcare professionals and new approaches to public policy to reduce breast cancer disparities.

## Background

Breast cancer is the most common cancer and the second highest cause of cancer death among women, exceeded only by lung cancer. Although it has among the highest 5-year rates of survival among cancers (90%, compared to 47% for ovarian cancer and 10% for cancer of the pancreas), it nonetheless affects a very high percentage of women. According to the National Cancer Institute, one in eight women, or 12.9% of all women, will develop breast cancer at some point in their lifetimes (2). The combination of high rates of survival and high rates of incidence means that a number of women will live with breast cancer for significant periods of their lives.

Differences in incidence, mortality, and survival occur by race and ethnicity. The National Cancer Institute's Surveillance, Epidemiology, and End Results (SEER) program and the Centers for Disease Control and Prevention's National Program of Cancer Registries have followed incidence and survival trends by Black and white race since 1975, with data on Asian/Pacific Islander, Hispanic/Latina, and American Indian/Alaska Native subpopulations added in 1990. Yet, studies of incidence and mortality have principally focused on race alone (i.e., without also considering ethnicity), and have found marked differences in both incidence and mortality.

DeSantis et al. analyzed SEER incidence data on breast cancer from 2012 to 2016 and SEER mortality data from 2013 to 2017 (3). They found that incidence rates were highest among white women (130.8 per 100,000), followed closely by Black women (126.7 per 100,000). Incidence was lowest among Asian/Pacific Islander women at 93.2 per 100,000. The picture differs for breast cancer mortality, in which the rates for Black women are 40% higher than those of white women (28.4 per 100,000 and 20.3 per 100,000, respectively), and both higher than other groups. The rates for Asian/Pacific Islander women, for example, were lower than those of either white or Black women (11.5 per 100,000).

When age is considered, additional differences between Black and white women emerge. Black and white differences in breast cancer mortality are most pronounced at younger ages and begin to converge later in life. Black women 50 years of age and younger, for example, are 1.9–2.6 times more likely to die from breast cancer than white women of the same age, yet they are only 1.1–1.2 times more likely to die from the disease at 70 years of age or older (3).

Mortality differences by age have in part been attributed to differences in the proportions of breast cancer molecular subtypes experienced (4). This is because mortality rates differ across breast cancer subtypes. For example, HR-positive/HER2 negative (hormone receptor-positive and human epidermal growth factor receptor 2) breast cancers, which have the most favorable outcomes, are 23% higher in white women over the age of 20 years than Black women of the same ages, and 45% higher than in Hispanic and American Indian/Alaska Native women of those ages. The triple-negative breast cancer (ER-negative, PR-negative, and HER2-negative) subtype, for which outcomes are the least favorable, is more common among Black women 50 years of age and younger. Thus, there is an interplay between race, age, and breast cancer subtype. Yet, this does not explain the mortality differences between Black and white women.

More recent efforts that have considered both ethnicity and race have yielded a more nuanced picture that may shed some additional light on disparities. Davis-Lynn and colleagues used SEER data to examine trends for non-Hispanic women vs. Hispanic women (5). The authors found that while Black and white women's incidence rates began to converge in 2012, the picture differs somewhat when ethnicity is added to analyses. In their study, incidence rates were highest for non-Hispanic white women and lowest for Hispanic white women, with non-Hispanic Black women's rates in between the two. This split between non-Hispanic and Hispanic white women, with non-Hispanic Black women between the two, provides additional nuance to our understanding of how race and ethnicity contribute to breast cancer disparities.

## Social Determinants and Breast Cancer

In addition to the effects of age and molecular effects, it is now widely recognized that social determinants of health such as racism, racial residential segregation, economic hardship, and housing insecurity, drive the production of racial/ethnic health inequities in the United States (6). Further, the United States does not provide universal health insurance to its citizens and residents. Uninsurance and underinsurance have been associated with poorer health outcomes including later disease detection, poor medication adherence and management of chronic illnesses (7). Specifically, lack of health insurance is associated with later stage of breast cancer diagnosis among Black, Indigenous, and Latinx women compared to white women (8). Other structural features of the United States including the implementation of racist policies and practices, such as redlining, contribute to the country remaining deeply segregated by race. Most health-promoting resources, such as access to healthful food options, safe places to recreate, and healthcare, are patterned by race. While an in-depth discussion of the policies and practices that led to the extraordinarily high, deeply entrenched racial residential segregation throughout the country is beyond the scope of this paper, it is important to note that segregation did not occur naturally. Rather, segregation was “by design” (9).

However, the highly impactful, insidious nature of segregation must be underscored (10). Most of the health-promoting resources, both directly and indirectly related to health, are afforded by context. Therefore, many Black Americans reside in neighborhoods that prohibit them from achieving their optimal level of health, including obtaining breast cancer screening and treatment (11). These context inequities are linked to poorer breast cancer outcomes among Black women.

Socioeconomic status (SES), including vital resources such as education, income, and wealth, is another key social determinant of health. For example, researchers have highlighted the overall importance of education, not only health literacy and communicating effectively with providers, but providing access to the higher levels of income and stable employment with benefits. Income is critical in helping individuals to afford their day-to-day needs such as food, housing, and services. More income allows individuals to purchase homes in more desirable, better-resourced neighborhoods. In this way, researchers have described SES as a fundamental cause—allowing individuals to avoid health risks. Employment is also important, especially since most Americans obtain their health insurance through their employer (12). In addition to healthcare insurance, paid time off, and the flexibility in work schedules to take the time to obtain screening or adhere to treatment plans are other critical benefits associated with the types of jobs individuals can access. Because Black women develop breast cancer at a younger age than white women, they are more likely to be diagnosed prior to retirement than white women, and thus to rely on employer-provided health insurance. Otis Brawley tells the poignant story of a young Black woman diagnosed with breast cancer who was the sole provider for her small children (13). She died because although she had health insurance through her employer, she lacked sufficient sick days to accommodate the treatment regimen recommended by her oncologist. Her difficult choice to keep her job to provide for her children cost her life.

In support of the contribution of health insurance to breast cancer mortality, is recent evidence of the effect of Medicaid expansion on rates of screening mammography. Screening mammography is important because if breast cancer is diagnosed early, treatment can begin before cancer cells have proliferated. Toyoda et al. found that mammography screening rates were significantly higher in states that expanded Medicaid than states that did not (14). Le Blanc et al. examined breast cancer stage, race/ethnicity, age, and insurance status using SEER data from 2007 to 2016. This allowed a comparison before and after the 2010 passage of the Affordable Care Act, which gave states the option of expanding Medicaid (15). The authors found that Medicaid expansion was associated with reduced incidence of advanced breast cancer, with Black women and women under 50 years of age achieving the greatest benefit. The incidence rates of Black women in expansion states decreased from 24.6 to 21.6%, compared to 27.4–27.5% in states that did not expand Medicaid (15).

There are policies and practices beyond healthcare that have influenced breast cancer inequities. The cleavages of Jim Crow policies, state and local laws that were adopted to oppress Black Americans and enforce segregation, continue to manifest today, including in breast cancer outcomes. For example, Krieger et al. examined whether breast cancer outcomes differed by birth in a Jim Crow state. They found that Black women who were born in Jim Crow states had poorer breast cancer outcomes, including more aggressive forms of cancer, compared to white women, regardless of their state of birth (16).

Health inequities, such as racial differences in breast cancer mortality, are strongly influenced by neighborhood context, including access to health promotive resources such as full-service grocery stores and safe places to recreate, in addition to preventative healthcare (10). Social environmental stressors, especially chronic exposure to these stressors, play a critical role in racial/ethnic breast cancer inequities. Researchers have documented the extent to which chronic stressors are deleterious to human health through their activation of a cascade of physiological reactions such as the release of hormones. Yet, the same adaptive mechanisms that allow individuals to escape life-threatening situations, such as increased blood pressure, are associated with poor health outcomes when activated chronically. Chronic activation of the physiological stress response system is associated with poor immune system functioning, which could contribute to negative cancer outcomes (17–20).

As technology and analysis tools are refined, researchers are better able to delineate the effects of the social environment on cancer incidence and the social patterning of cancer. For example, scholars have demonstrated the role of epigenetics in the occurrence of cancer and differential vulnerability to cancer across race/ethnicity (21). Epigenetic changes, or those that occur through changes in how genes are expressed rather than through changes in underlying gene sequence, represent a potential route through which the social environment affects physiological responses. Linnenbringer et al. link this to breast cancer mortality disparities by suggesting that weathering (i.e., wearing down over time) of the body's stress response system may contribute to the expression of breast cancer subtypes with less favorable outcomes (22).

**Figure 1:**
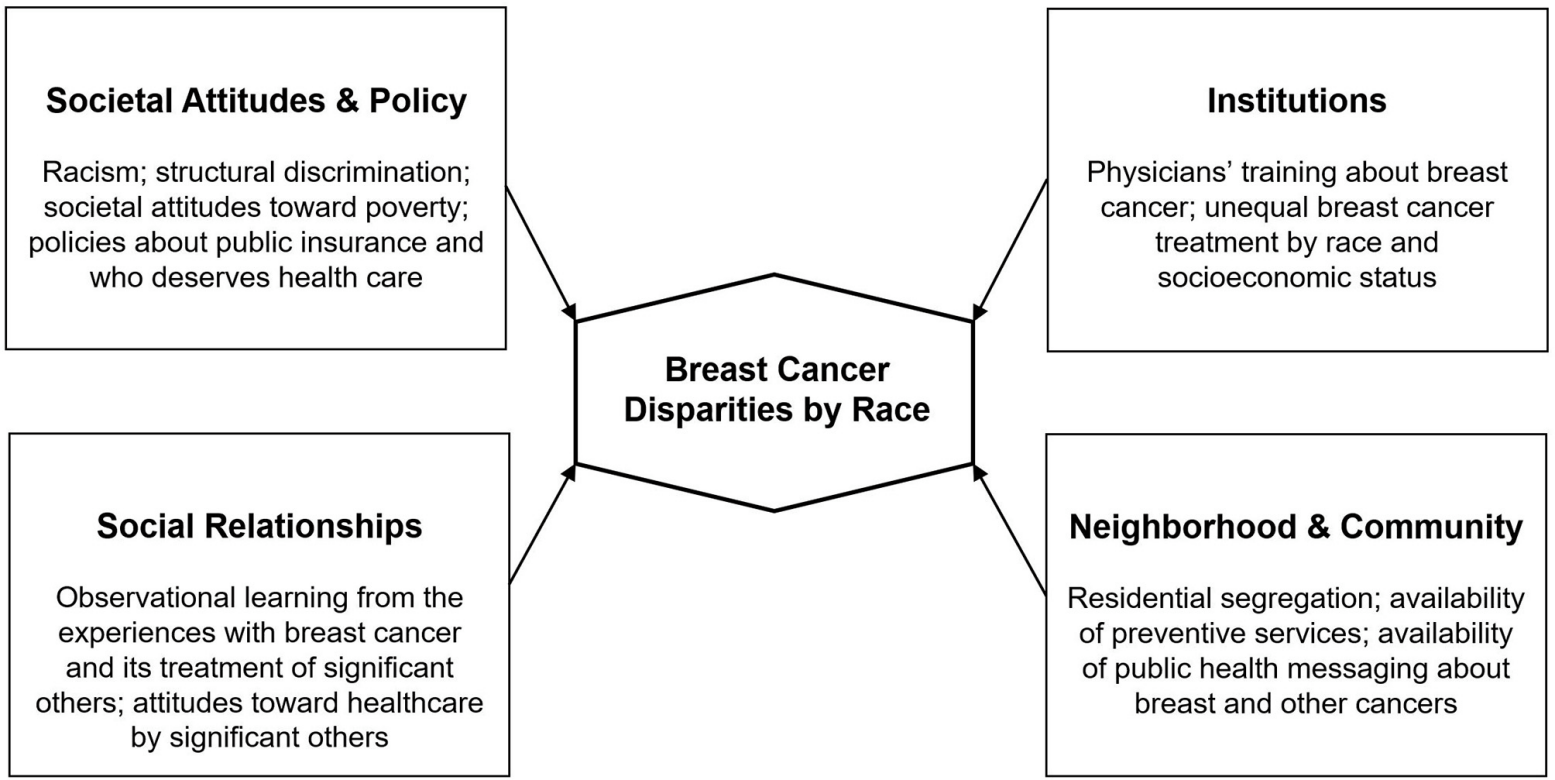
Social factors that influence differential breast cancer disparities by race.

It is clear that without addressing the barriers imposed by social determinants of health such as racism, housing stability, and access to quality education, it is highly likely that observed racial disparities in breast cancer will persist, even as screening and treatment improve [(23, 24); see Figure 1].

## Healthcare Inequities

In addition to the aforementioned social determinants of health, the United States health system presents unique barriers to care as well as deeply entrenched biases based on race, gender, immigration status, among other factors. A seminal study on racial bias in healthcare, *Unequal Treatment: Confronting Racial and Ethnic Disparities in Health Care*, found that racial and ethnic minorities received less than the standard of care across many health conditions, including heart and kidney care and health services including intensive care (25). Racial inequities were found even after controlling for income, insurance status, and age (25). The authors also concluded that provider-side bias and patient mistrust contribute to differences in treatment (25).

More recent studies have found that Black people continue to experience inequities in healthcare treatment, above and beyond those that come from barriers to access. For example, a large body of evidence indicates that Black people are systematically undertreated for pain compared to white people (26, 27). Further, a study of over 400 medical students and residents found that differences in pain management could be explained by the fact that some providers believe Black people are different biologically than white people (26). Hoffman et al. found that a large number of white laypeople, medical students, and residents believe a constellation of erroneous beliefs about Black people's biology including that Black people have thicker skin, fewer nerve endings, and smaller brains. Importantly, these erroneous beliefs predict the accuracy of the providers' pain management recommendations. The study is among the first to demonstrate that false beliefs about biological differences between racial groups directly affect treatment.

Racial and ethnic differences in post-mastectomy pain management have also been found. A study of over 80,000 women who had undergone mastectomies reported Black, Latinx, and Asian women were less likely to receive regional anesthetic techniques, i.e., focused pain relief at the surgical site, than white women (28). This is in spite of the fact that regional anesthetic techniques are increasingly favored for the management of post-mastectomy pain. Pain from treatments, either experienced personally or described by trusted others, may act as a deterrent to participation in screening and treatment.

Breast cancer screening is effective in detecting breast cancer early, thus allowing for earlier treatment to prevent progression (29). Recent evidence using SEER data demonstrates that when factors other than race are controlled for, no significant Black and white differences in screening mammography rates are found (30). The Centers for Disease Control and Prevention (CDC) instead found lower screening rates to be associated with lower educational attainment and income, not having a usual source of care, and being uninsured or having only public health coverage (30). Thus, it seems that Black women being diagnosed later than their white counterparts has more to do with social determinants than with anything inherent in race.

All told, racial and ethnic minorities in general, and Black women in particular, continue to face discrimination and inequities in treatment. Despite findings that indicate that Black women have similar rates of breast cancer screening as white women are more compliant with breast cancer screening recommendations, they are still more likely to experience more aggressive, rapidly advancing cancer compared to white women (31). As a result, mistrust of healthcare providers and medical institutions may be understood as a rational adaptation to a healthcare system that is often implicitly and explicitly hostile to Black people. The research literature suggests Black and Latinx people, including Black women, are less likely than white people to trust their physician, even after controlling for socioeconomic status, health status, and healthcare access (32).

## Key Gaps in Knowledge and Action

### (Mis)conceptualizing Race

To reduce disparities in breast cancer mortality, cancer researchers and practitioners may benefit from the application of Critical Race Theory (CRT). One tenet of CRT is to examine race as a social factor rather than an immutable biological factor (16, 33, 34). As the aforementioned study by Hoffman notes, erroneous beliefs about innate biological differences between Black and white people contribute to differences in treatment (26). Historians and sociologists of science have demonstrated that much of medical practice rests on long-held, debunked beliefs about the fundamental differences between Black and white bodies. Steven Jay Gould, for example, wrote of the fundamental errors inherent in Morton's 1839 *Crania Americana*, in which Morton filled the crania of skulls with pepper seeds and equated cranial-capacity with intellectual ability. Morton interpreted differences across groups as evidence of the mental superiority of Caucasians (35, 36). In a second example, Lundy Braun relates that during slavery, Black people were believed to have poorer lung capacity compared to white people (37). Importantly, doctors did not consider the profoundly deleterious physical and psychological effects of slavery. Instead, they attributed health problems to the innate biological inferiority of Black people. Based on these false beliefs, modern spirometry meters were created to “correct for race” when no innate biological lung differences actually exist. Racial differences are, however, based on the social determinants of health, not innate physiological differences. However, Chowkwanyun and Reed argue that in spite of a well-documented cross-disciplinary critique of biological explanations of socially determined racial differences, this type of thinking persists in contemporary medical treatment (38).

To address the negative consequences of relying on biological definitions of race, some American medical students have organized to change the nature of medical training. In their report, *Toward the Abolition of Biological Race in Medicine*, Chadha et al. argue that racism, not race, causes health disparities (39). Further, because clinical training relies on biological explanations of racial differences, patients of color are systematically misdiagnosed and undertreated. In this manner, Chadha et al. point out that biological explanations of racial disparities in health fail to address structural discrimination. Brown University medical students have also noted that “preclinical medical curricula inaccurately employ race as a definitive medical category without context, which may perpetuate misunderstanding of race as a bioscientific datum, increase bias among student–doctors, and ultimately contribute to worse patient outcomes” [(40), p. 1]. Importantly, Tsai and colleagues reported that in response to the students' concerns, the medical school changed the curriculum to include a longitudinal race-in-medicine component (40). As such, to improve health and health care outcomes, we must refine medical training to root out both racial bias and the over-reliance on race over racism as a risk factor for illness.

To that end, researchers have begun to reconsider how race and racism should be factored into health disparities research. Increasingly, some scholars emphasize the need to move away from the idea of race as a risk factor for disease, which reifies the notion of race as a matter of biology, while turning to more structural explanations that center the ways racism harms human health (41). Boyd et al. argue that “racism kills. Whether through force, deprivation, or discrimination, it is a fundamental cause of disease and the strange but familiar root of racial health inequities” (41). They also note that despite recent calls to actively acknowledge structural racism as a determinant of health, the majority of health disparities research often defaults to genetic and/or biological explanations of racial differences in health outcomes (41).

### Policy Perspectives on Reducing Breast Cancer Disparities

CRT encourages scholars to move beyond the consideration of individual risk factors in the production of disease. Rather, an accurate sociohistorical perspective is necessary to fully understand inequities. Another tenet of CRT is to privilege the voices of marginalized people (16, 33, 34). This is critical in the development of policies that are capable of improving the environmental and social factors that Black women face and fuel inequities in breast cancer outcomes.

Metzl and Hansen have called for extending a structural lens to medical training and practice (42). They define structural competency as “the trained ability to discern how a host of issues defined clinically as symptoms, attitudes, or diseases (e.g., depression, hypertension, obesity, smoking, medication adherence, trauma, and psychosis) also represent the downstream implications of a number of upstream decisions about such matters as health care and food delivery systems, zoning laws, urban and rural infrastructures, medicalization, or even about the very definitions of illness and health” [(42), p. 128]. They also outline five key tenets of training medical providers: (1) recognizing the structures that shape clinical interactions; (2) developing an extra-clinical language of structure; (3) rearticulating “cultural” formulations in structural terms; (4) observing and imagining structural interventions; and, (5) developing structural humility. The authors advocate for helping medical practitioners to understand the ways in which socioeconomic forces contribute to epigenetic changes. This should be integrated with existing medical models to foster pedagogical change.

Given the body of research on weathering and critical periods in the potential development of breast cancer later in life, medical practice and social policy must be aligned to address the temporal, social-emotional, and physical needs of women, particularly ethno-racial minorities. Further, addressing systematic racism must be central to any strategy to reduce racial health disparities including breast cancer. Policy strategies may include: (1) expanding social safety net policies to improve social determinants of health and (2) addressing medical training to include structural competency, which emphasizes structural discrimination as opposed to biological explanations of socially patterned racial differences in health (36).

Based on state-level comparisons on social service expenditures (including cash transfers, food stamp benefits), Bradley and colleagues found that states that spent more on social services had better health outcomes (e.g., adult obesity, lung cancer mortality, mentally unhealthy days, type 2 diabetes) than states that spent less (43). Studies that evaluate the relationship between social spending and health outcomes in OECD (Organization for Economic Cooperation and Development) countries compared to the United States find similar patterns (44).

The above studies suggest that investing in social safety net programs may improve health outcomes overall, particularly for racial and ethnic minorities who are disproportionately represented among people living in poverty. To that end, Newman et al. outline several policy recommendations to mitigate inequities in health that are germane both to cancer disparities and those that have emerged from the current COVID-19 pandemic (45). They argue that a combination of community collaboration, increased racial diversity in clinical trials, expanding health insurance, and increased funding for safety-net hospitals, would go a long way toward mitigating both cancer and COVID-19 racial health inequities.

All told, recent scholarship suggests a move away from individual-level interventions toward more macro-level policy solutions. Conceptualizing cancer disparities at the structural level provides an important framework for moving forward in this vein. It is not until this occurs that we can begin to see health equity.

### Dissemination and Implementation

CRT guides scholars to affirm the knowledge of Black people, privileging the voices of those who have experiential knowledge of being marginalized in order to highlight to where interventions should be directed (33). This calls for an intentional consideration of the experiences Black women in order to craft appropriate strategies to redress breast cancer disparities.

One way to facilitate the dissemination of information about breast cancer is to use storytelling and narrative. Qualitative research is a powerful tool to elucidate the barriers that Black women face related to breast cancer screening and treatment seeking (46, 47). Narratives aid in advocacy efforts in a way that is often more powerful than simply displaying data. A broader application of this aspect of CRT is through meaningful community engagement. The principles of community engagement can aid in privileging historically marginalized voices to address racial health inequities (48, 49). For example, by building the capacity of organic social networks that exist within communities and providing linkages between communities and other sectors, such as business and government, communities can gain greater collective efficacy, setting the agendas and goals needed to advocate for needed resources (50).

## Discussion

Black-White inequities in breast cancer are well-established. These observed racial inequities are driven more by social, environmental, and economic factors than by biological factors (51, 52). As scholars and practitioners consider ways to narrow Black-white inequities, it is critical to examine the structural factors that are both determinants of breast cancer as well as barriers to screening and care (53). We implore the field to delve deeper, beyond rudimentary “racial” explanations and individual risk factors to consider the broader ecology in which people are embedded. This requires an understanding of the key factors that have shaped their environments, both to reduce victim-blaming and to motivate new solutions to the barriers faced by historically marginalized communities. It is critical that more robust health promotion efforts are developed to promote cancer screening and navigate complex treatment environments. Engaging with a range of communities will help to ensure that health communication messaging and promotion efforts are calibrated to the needs of Black women. Effectively building that knowledge base and crafting appropriate solutions will require amplifying, validating, and incorporating the voices of these communities, all of which are critical to any effective policy or practice change efforts (54).

## Data Availability Statement

The original contributions presented in the study are included in the article/supplementary material, further inquiries can be directed to the corresponding author/s.

## Author Contributions

SG, DH, and TS: conceptualization and writing. All authors contributed to the article and approved the submitted version.

## Conflict of Interest

The authors declare that the research was conducted in the absence of any commercial or financial relationships that could be construed as a potential conflict of interest.
